# *Ab Initio* Prediction of the Phase Transition for Solid Ammonia at High Pressures

**DOI:** 10.1038/s41598-020-64030-3

**Published:** 2020-05-05

**Authors:** Lei Huang, Yanqiang Han, Jinyun Liu, Xiao He, Jinjin Li

**Affiliations:** 1 Key laboratory for Thin Film and Microfabrication of the Ministry of Education, Department of Micro/Nano-electronics, Shanghai Jiao Tong University, Shanghai, 200240 China; 2 Shanghai Engineering Research Center of Molecular Therapeutics and New Drug Development, School of Chemistry and Molecular Engineering, East China Normal University, Shanghai, 200062 China; 3 Key Laboratory of Functional Molecular Solids of the Ministry of Education, Anhui Laboratory of Molecule-Based Materials, College of Chemistry and Materials Science, Anhui Normal University, Wuhu, Anhui 241000 China; 4 NYU-ECNU Center for Computational Chemistry at NYU Shanghai, Shanghai, 200062 China

**Keywords:** Phase transitions and critical phenomena, Theoretical chemistry

## Abstract

Ammonia is one of the most basic components on the planet and its high-pressure characteristics play an important role in planetary science. Solid ammonia crystals frequently adopt multiple distinct polymorphs exhibiting different properties. Predicting the crystal structure of these polymorphs and under what thermodynamic conditions these polymorphs are stable would be of great value to environmental industry and other fields. Theoretical calculations based on the classical force fields and density-functional theory (DFT) are versatile methods but lack of accurate description of weak intermolecular interactions for molecular crystals. In this study, we employ an *ab initio* computational study on the solid ammonia at high pressures, using the second-order Møller-Plesset perturbation (MP2) theory and the coupled cluster singles, doubles, and perturbative triples (CCSD(T)) theory along with the embedded fragmentation method. The proposed algorithm is capable of performing large-scale calculations using high-level wavefunction theories, and accurately describing covalent, ionic, hydrogen bonding, and dispersion interactions within molecular crystals, and therefore can predict the crystal structures, Raman spectra and phase transition of solid ammonia phases I and IV accurately. We confirm the crystal structures of solid ammonia phases I and IV that have been controversial for a long time and predict their phase transition that occurs at 1.17 GPa and 210 K with small temperature dependence, which is in line with experiment.

## Introduction

Ammonia is an attractive energy carrier because of its high energy density and potential as a “zero carbon” fuel^1,2^. Owing to the high vapor pressure and toxicity of liquid ammonia, solid ammonia provides a solution for liquid ammonia storage. Solid ammonia can often pack in multiple distinct polymorphs with different crystal structures and physical properties^3–6^. For example, the solid ammonia phase I (with a space group of *P2*_1_3)^3,7,8^ has a cubic unit cell structure and the molecules are ordered, while in hexagonal close-packed phase II (with a space group of *P6*_3_*/mmc*)^5^ and face-centered cubic phase III (space group *Fm3m*)^4,9^, the molecules are disordered. Further increasing the pressure generates a new ordered solid phase IV, which stabilizes over a wide range of pressure up to 70 GPa^10^.

Due to the limitations of experimental conditions and the wide range of existence in the phase diagram, the crystal structure of solid ammonia phase IV has been debated for a long time. For example, early X-ray powder diffraction study performed by Gauthier *et al*.^10^ found that solid ammonia phase IV is a disordered hexagonal close-packed (hcp) structure (has a space group of *P63/mmc*) with four molecules per unit cell. But such conclusion has been disputed by Loveday *et al*.^6^, where they determined the crystal structure of phase IV with a space group of *P2*_1_2_1_2_1_ through high quality neutron diffraction patterns, and such result has been confirmed by Fortes *et al*.^11^ via the plane-wave pseudopotential calculation. Raman spectrum is also a powerful tool to investigate the crystal structures and to distinguish different polymorphs of a material. As early as 1988, Gauthier *et al*.^10^ observed five major vibrational modes from 100 to 500 cm^−1^ and three modes from 3,000 to 3,500 cm^−1^ in the Raman spectrum of solid ammonia sample at room temperature. However, a few years later, Kume *et al*.^12^ measured seven and eight Raman vibrational modes in those two frequency regions, respectively. In Kume *et al*.’ s study^12^, the solid ammonia phases I and IV were measured to be pseudo-close-packed structures with very small free energy difference. They believed that the thermal energy can activate the phase transition between phases I and IV, and their phase transition was speculated to occur at low temperatures. It is therefore of relevance to chemical physics to confirm such speculation and to understand the nature of hydrogen bonding in crystalline ammonia.

Given the high cost of discovering novel materials and performing experimental solid-phase screening, many studies have focused on the computational structure predictions^13–18^. Fortes *et al*.^11^ carried out *ab initio* simulations of solid ammonia phases I and IV under high pressures, based on the plane-wave pseudopotential calculations, where they predicted the lattice constants and equations of state but failed to reproduce the phase transitions between phases I and IV owing to the limited accuracy when treating the hydrogen bonding interactions within solid ammonia. In the past few decades, electronic structure methods have made great progress in predicting the molecular crystal energies and optimizing the crystal structures. Progress in three main directions is primarily responsible for the significant improvement: (1) the density function theory (DFT) that includes van der Waals dispersion interactions^19–22^, (2) the second-order Møller-Plesset perturbation (MP2) theory^23–26^, which considers the hydrogen bonding, ionic, covalent and dispersion energies, (3) fragment-based quantum mechanical (QM) methods which greatly reduces the computational cost of electronic structure calculations for large systems^27–29^. On the one hand, DFT has become a standard method for handling the systems of liquids and solids, but its accuracy relies on the selection of density functional, which cannot be systematically improved by any general procedure. On the other hand, there have been a number of developments in fragment-based electronic structure methods that have greatly expanded the applications of correlated wave function methods (e.g., MP2 theory, coupled cluster singles, doubles, and perturbative triples (CCSD(T)) into biological and other large molecules^30–34^.

The fragment-based QM approach increasingly reduces the computation time with high level wavefunction theories and enables the high accuracy calculations in theoretically challenging questions. The present work investigates the lattice constants, equations of state, Raman spectra, Gibbs free energies of solid ammonia phases I and IV using the MP2 theory, and thus predicts their phase transitions at high pressures and finite temperatures. To calculate the Gibbs free energy of solid ammonia efficiently and accurately, we use the Electrostatically Embedded Generalized Molecular Fractionation (EE-GMF)-based MP2 theory^25,35–39^ in this study. The EE-GMF is an embedded fragment QM method, which can treat the macromolecules effectively^31^ by decomposing the internal energy of the unit cell of the crystal into the energy combination of monomers and dimers. These monomers and dimers are embedded in the electrostatic field of the rest of the crystalline environment. In a certain distance threshold, the interaction energy between two fragments is calculated by quantum mechanics, and the interaction between two long-range fragments is calculated by charge-charge Coulomb interaction. The structures, equations of state and solid-solid phase transition of I-IV are systematically studied and compared over the range of 0–220 K and 0–4 GPa, as well as Raman spectra of phases I and IV. Rather than classical force field method and density-functional theory (DFT)^19–22^, MP2 has an ability to describe hydrogen bonding, ionic, covalent, and dispersion interactions efficient, and therefore can predict the crystal structures and phase transitions accurately^40–43^.

## Methods

### Free energy calculation

The Gibbs free energy, *G*, of a molecular crystal at temperature *T* is defined by^44^,

1 $${\rm{G}}=E+PV+Uv-TSv$$

where *E*, *V*, *U*_*v*_ *S*_*v*_ are the internal electronic energy, volume, zero-point vibrational energy and vibrational entropy per unit cell at temperature *T*, respectively.

To calculate the internal electronic energy of the molecular crystal, we apply the EE-GMF method^25,35–39^ and assign each individual ammonia molecule as a fragment. The internal electronic energy includes the energy of each fragment, the two-body interaction energy between two fragments that are spatially in close contact and the interaction energies between two distant fragments. The former are computed by the QM method, while the latter are evaluated via pairwise charge-charge Coulomb interactions. According to the EE-GMF method, the energy per unit cell can be expressed as follows^38,39^,

2 $$\begin{array}{lll}{E}_{{\rm{cell}}} & = & \sum _{i}{\tilde{E}}_{i(0)}+\sum _{\begin{array}{c}i,j,i < j\\ |{{\bf{R}}}_{i(0)j(0)}|\le \lambda \end{array}}({\tilde{E}}_{i(0)j(0)}-{\tilde{E}}_{i(0)}-{\tilde{E}}_{j(0)})+\frac{1}{2}\mathop{\sum }\limits_{n=-S}^{S}(1-{\delta }_{n0})\\  &  & \times \sum _{\begin{array}{c}i,j\\ |{{\bf{R}}}_{i(0)j(n)}|\le \lambda \end{array}}({\tilde{E}}_{i(0)j(n)}-{\tilde{E}}_{i(0)}-{\tilde{E}}_{j(n)})-{E}^{{\rm{DC}}}+{E}^{{\rm{LR}}}\end{array}$$

where *n* is the index of a unit cell, *S* is the number of neighboring unit cells treated by QM, *E*_*i*(*n*)_ is the energy of the *i*th molecule in the *n*th unit cell and *E*_*i*(0)*j*(*n*)_ is the energy of the dimer consisting of the *i*th molecule in the central (0th) unit cell and the *j*th molecule in the *n*th unit cell. The QM energy calculations of monomer *i*(0) and dimer *i*(0)*j*(n) are performed in the embedded electrostatic field of the rest of the system, which are represented by Coulomb field of atomic charges in order to account for the electronic polarization effect from the surrounding environment. $\tilde{E}$ in Eq. (2) denotes the sum of the self-energy of the fragment along with the interaction energy between the fragment and background charges of the remaining system.

The first term in Eq. (2) includes the energy of each molecule in the central unit cell, and the second term is the local two-body QM interactions if the nearest distance between any two molecules in the central unit cell is less than or equal to a predefined distance threshold λ (λ is set to 4 Å in this study)^37,45–48^. For the interactions between the central unit cell and the neighboring unit cells, we also calculate the local two-body QM interactions if any molecule in the central unit cell has close contact with other molecules in the neighboring unit cell, as expressed in the third term of Eq.(2). In the case that the nearest distance between two molecules is larger than λ, these long-range interactions are approximately described through charge-charge Coulomb interactions (*E*^*DC*^, which is doubly counted in the previous three terms of Eq.(2)^37,45–48^).

All QM calculations are performed at the MP2/aug-cc-pVDZ level, in the electrostatic field of the rest of the crystal represented by the electrostatic potential (ESP) charges self-consistently determined through the EE-GMF approach at the HF/aug-cc-pVDZ level. We treat the dimers of 3 × 3 × 3 unit cells (i.e., *S* = 1 in Eq. 2) quantum mechanically, in which the distance between the dimer is within the distance threshold λ. The background charges are included within the 11 × 11 × 11 supercell. In addition, *E*^LR^ in Eq. (2) represents the long-range interactions within the 41 × 41 × 41 supercell through Coulomb interactions for the central unit cell. All QM calculations are carried out using the Gaussian09 program^49^.

For a molecular crystal, the zero-point vibrational energy (*U*_*v*_) and vibrational entropy per unit cell (*S*_*v*_) can be obtained by Eqs. (3) and (4) with the harmonic approximation,

3 $${U}_{v}=\frac{1}{K}\sum _{n}\sum _{{\bf{k}}}{\omega }_{n{\bf{k}}}\left(\frac{1}{2}+\frac{1}{{e}^{\beta {\omega }_{n{\bf{k}}}}-1}\right)$$

4 $${S}_{v}=\frac{1}{\beta TK}\sum _{n}\sum _{{\bf{k}}}\left\{\frac{\beta {\omega }_{n{\bf{k}}}}{{e}^{\beta {\omega }_{n{\bf{k}}}}-1}-\,\mathrm{ln}(1-{e}^{-\beta {\omega }_{n{\bf{k}}}})\right\}$$

where *β* = (*k*_*B*_*T*)^−1^, *k*_B_ is the Boltzmann constant, and *ω*_*n***k**_ is the frequency of the phonon in the *n*th phonon branch with the wave vector **k**. The product over **k** must be taken over all *K* evenly spaced grid points of **k** in the reciprocal unit cell. In this study, the **k**-grid of 21 × 21 × 21 has been used (K = 9,261).

### Complete Basis Set (CBS) correction

In QM calculations, the accuracy of wavefunction-based methods depends on the reliability of the method used and the quality of the basis set. To approach the complete basis set (CBS) limit, large basis sets are needed and their practical applications are currently limited to relatively small molecular systems. Attempts to obtain the CBS results lead to various extrapolation schemes^50–53^, which approximate the CBS limit by extrapolating the results from finite basis sets. Two-point or three-point extrapolation formulas are employed in most of such extrapolation schemes, each of which is obtained from a growing base set.

In the present work, the CBS extrapolated value of MP2 correlation energy (*E*_MP2/CBS_) is obtained from two-point extrapolation scheme^51^.

5 $${E}_{{\rm{MP2}}/{\rm{CBS}}}={E}_{{\rm{MP2}},X}+{\rm{constant}}\times {X}^{-3}$$

where X represents the largest angular momentum of the given basis set, i.e., X = 2 for aug-cc-pVDZ (ADZ), and X = 3 for aug-cc-pVTZ (ATZ). The HF energies are not extrapolated and simply taken from the results of the ATZ basis set used in the given extrapolation scheme. The CBS correlation energies for CCSD(T) are obtained by^51,54–56^

6 $$\,{E}_{{\rm{CCSD}}({\rm{T}})/{\rm{CBS}}}={E}_{{\rm{MP2}}/{\rm{CBS}}}+({E}_{{\rm{CCSD}}({\rm{T}})/{\rm{ADZ}}}-{E}_{{\rm{MP2}}/{\rm{ADZ}}})$$

which is based on the observation that the difference between the MP2 and CCSD(T) correlation energies converges faster in basis set size than the correlation energies themselves.

## Results and discussion

### Equations of state

**Figure 1:**
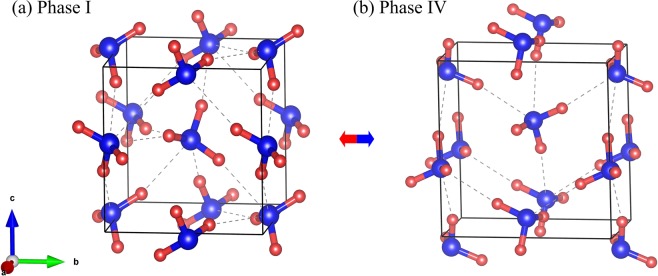
The crystal structures of solid NH_3_ phases I (**a**)^8^ and IV (**b**)^6^. The blue and red balls represent N and H atoms, respectively.

The crystal structures of solid ammonia phases I (with a space group of *P2*_1_3)^8^ and IV (with a space group of *P2*_1_2_1_2_1_)^6^ are shown in Fig. 1. For phase I, the cubic unit cell contains four orientationally ordered ammonia molecules on C_3v_ symmetry sites^8^. The dipole moments of the ammonia molecules point to the crystallographic [111] directions. Each molecule accepts and donates three hydrogen bonds, each of which is very different from the almost completely linear hydrogen bond. Since the three nearest neighbors share a single-strand pair of orbitals, the hydrogen bonds in ammonia solids are very weak, resulting in the filling of pseudo-fcc molecules [111]. The structure of solid ammonia phase IV is shown in Fig. 1(b), which also contains four ordered molecules but is an orthorhombic, not hexagonal close packed (hcp) unit cell structure. The nitrogen atoms have a pseudo-hcp arrangement. In the present study, we apply EE-GMF based MP2/aug-cc-pVDZ method to optimize their respective crystal structures. Detailed data are shown in Table 1. At 0.1 GPa, the calculated lattice constants of phase I are a = b = c = 4.923 Å, while at 5 GPa, the calculated lattice constants of phase IV are a = 3.142 Å, b = 5.599 Å, c = 5.445 Å, respectively.

**Table 1:** Observed and calculated lattice constants of ammonia phases I and IV.

Parameter	Expt. Phase I^8^ at 0 GPa, 2 K	MP2 Phase I at 0.1 GPa, 0 K	Expt. Phase IV^6^ at 5 GPa, 150 K	MP2 Phase IV at 5 GPa, 0 K
a/Å	5.048	4.923	3.250	3.142
b/Å	5.048	4.923	5.658	5.599
c/Å	5.048	4.923	5.356	5.445

**Figure 2:**
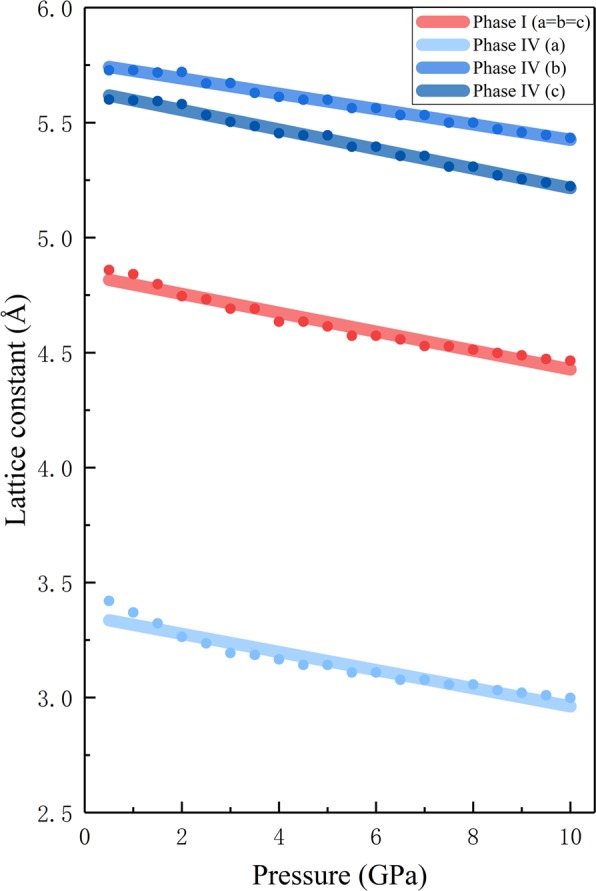
Pressure dependence of calculated lattice constants of solid phase I (a = b = c) and phase IV. The red line represents the lattice constants of phase I, while blue lines show lattice constants of phase IV. The lines between the dots are drawn to guide the eye.

**Figure 3:**
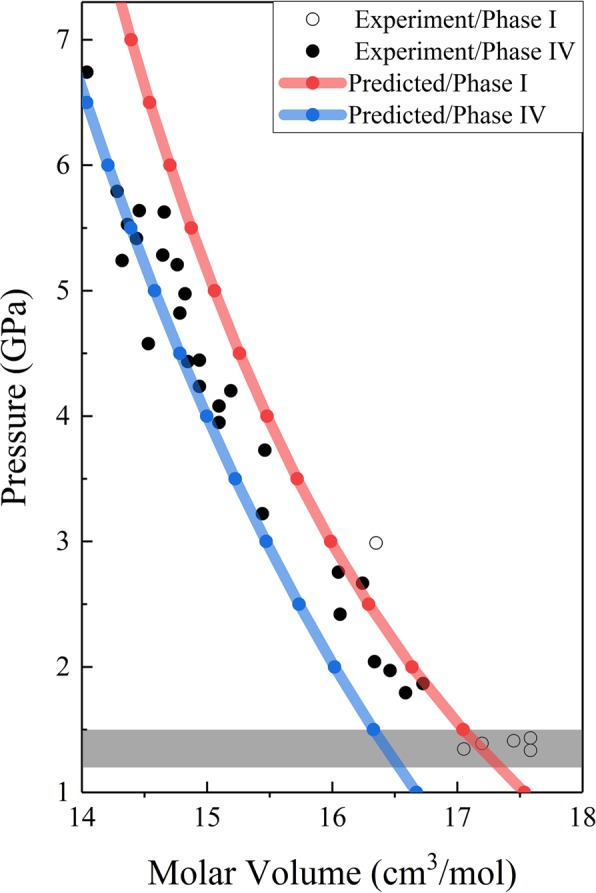
The calculated pressure dependence of unit cell volume for solid ammonia phases I (blue curve) and IV (red curve) along with the experimental data. The white and black dots denote the experimental volumes of phases I and IV, which are taken from Otto *et al*.^57^ and Loveday *et al*.^6^, respectively. The lines between the dots are drawn to guide the eye.

Figure 2 shows the calculated lattice constants of ammonia phases I and IV. For cubic phase I, we have a = b = c, while phase IV is an orthorhombic structure, which follows an ideal pseudo-hcp arrangement, and the axial ratios of phase IV are close to but differ from the ideal values $({\rm{b}}=\sqrt{3{\rm{a}}},{\rm{c}}=\frac{\sqrt{8}}{\sqrt{8}}{\rm{a}})$. The calculations predict smooth pressure dependence for both phases I and IV without noticeable discontinuity between 0–10 GPa at 0 K. Figure 3 shows the relationship between pressure and molar volume of NH_3_ phases I and IV, respectively, along with the experimental data^6,57^. It is worth noting that the predicted lattice volumes of phases I and IV, decreasing as the pressure increasing, are in good agreement with the observed results. The shade in Fig. 3 shows the volume reduction upon the transition from phase I to phase IV, which is also consistent with the experimental data.

### Vibrational spectra

**Figure 4:**
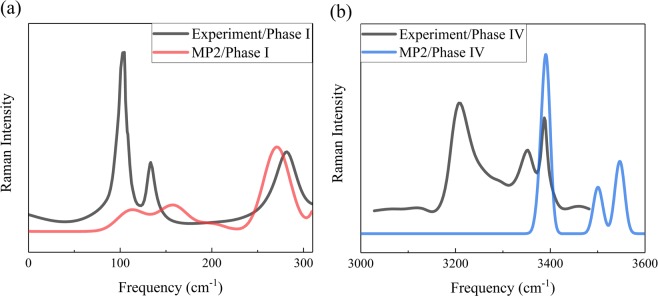
The comparisons of observed and calculated Raman spectra of solid ammonia phases I and IV. The black curves are the experimental Raman spectra that are taken from Binbrek *et al*.^58^ and Kume *et al*.^12^, while the red and blue curves are the calculated Raman spectra at 0.4 and 8.0 GPa, respectively, based on the EE-GMF-MP2/aug-cc-pVDZ method.

Raman spectrum serves as a fingerprint to identify the configurations of molecular crystals. The position and intensity of the Raman absorption peaks reflect the structural characteristics of molecules and can be used to determine the structural compositions and chemical groups. Figure 4(a,b) show the comparisons of calculated and observed Raman spectra of phases I and IV, respectively. For phase I, Binbrek *et al*.^58^ assigned three Raman peaks located at 103, 134 and 282 cm^−1^ (the black curve in Fig. 4(a)), while the MP2 calculations reproduce these three vibrational peaks with slight shifts (the red curve in Fig. 4). The calculated Raman spectra of phase IV are shown in Fig. 4(b), where the peak shifts between the calculated Raman peaks and observation come from the stretching motion of O-H bonds. The black curve in Fig. 4(b) is the experimental Raman spectrum of phase IV^12^, which displays three distinct Raman peaks located at 3,206.9, 3,352.5 and 3,389.5 cm^−1^, respectively. The MP2/aug-cc-pVDZ theory reproduce these three peaks, as shown in the blue curve of Fig. 4(b). Therefore, the present calculations reproduce the Raman spectra of ammonia phases with good accuracy.

**Figure 5:**
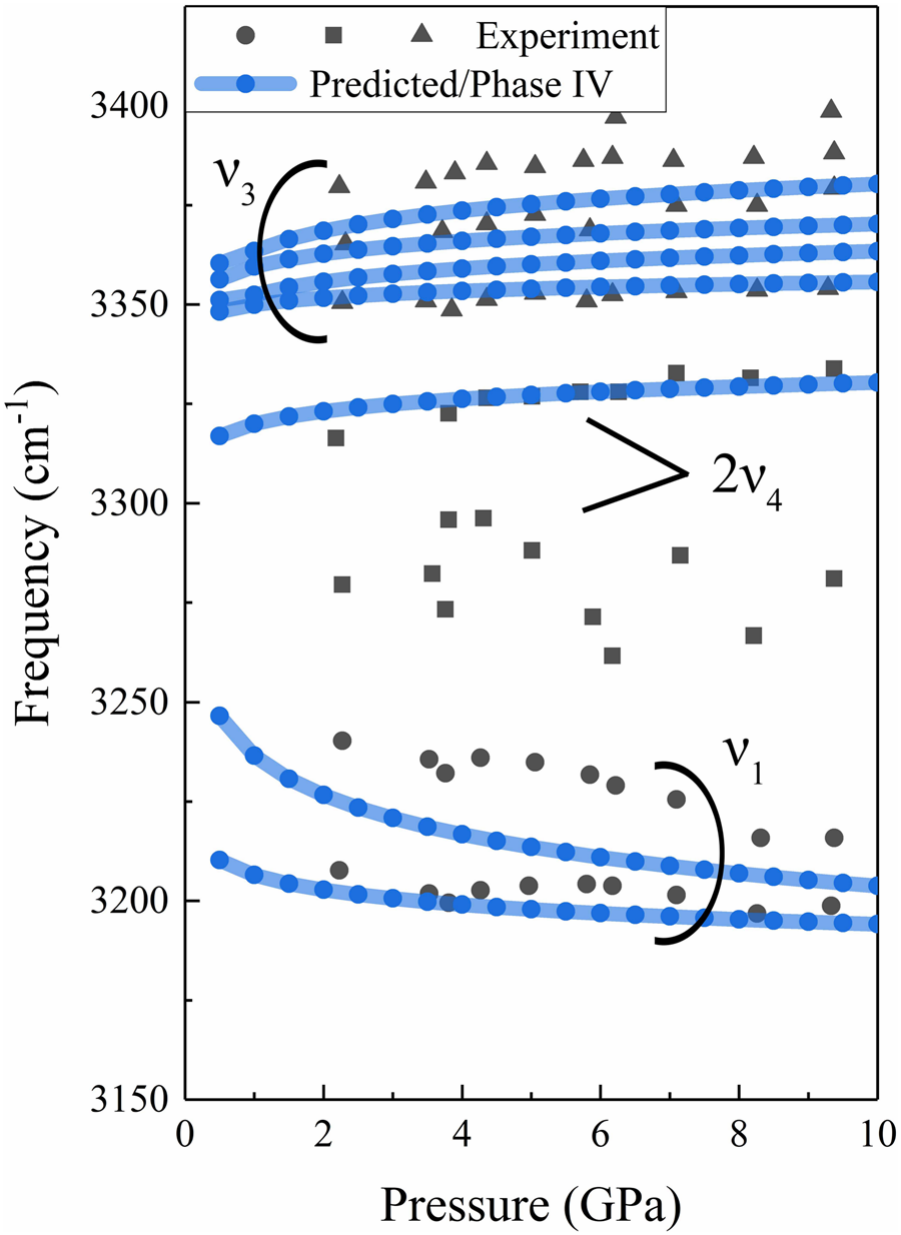
The Raman frequencies as a function of pressure, the black dots are experimental data from Kume *et al*.^12^ The lines between the dots are drawn to guide the eye.

Figure 5 plots the peak frequencies of the Raman bands at high frequency region, which can be separated into three modes, i.e., ν_1_, ν_3_ and ν_4_. The two bands located around 3,200–3,250 cm^−1^ (solid circles), assigning to ν_1_ mode in experiment, decrease with the increasing of pressure. The increasing pressure leads to the decreasing of intermolecular distance and the increasing of the intermolecular bond (hydrogen bond) strengths, which will decrease the internal covalent bonds and the internal stretching frequency. Therefore, the two bands around 3,200–3,250 cm^−1^ can be assigned to the symmetric stretching (ν_1_) modes. According to the experimental data from Kume *et al*.^12^, the four bands at high frequency region (the solid triangles in Fig. 5) are assigned to ν_3_ modes, which related to the Fermi resonance. In addition, the present study also reproduces the overtone mode ν_4_, which locates around the frequency at 3240 cm^−1^. Our predicted Raman frequencies is consistent with these 3 modes, and confirmed that the structure of phase IV is orthorhombic with a space group of *P2*_1_2_1_2_1_.

### Gibbs free energy

**Figure 6:**
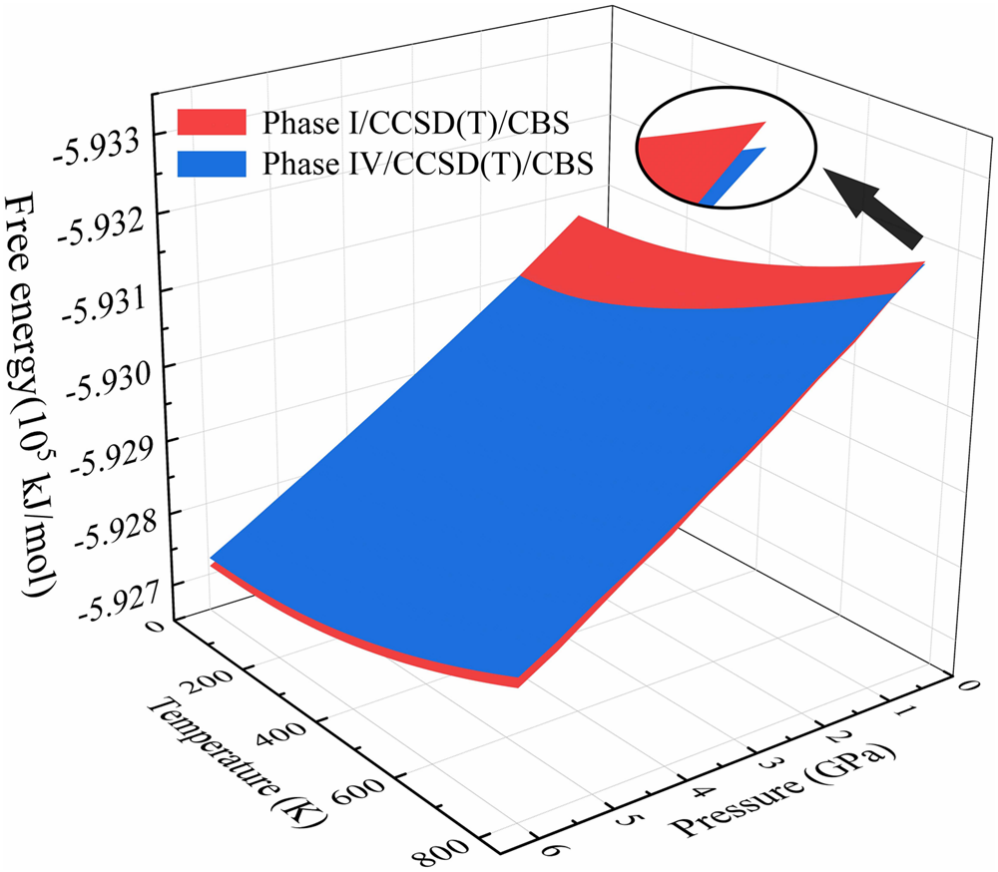
Gibbs free energy surfaces of solid ammonia phase I (red) and phase IV (blue) as functions of temperature and pressure. The Gibbs free energies are calculated by CCSD(T)/CBS.

To predict the stabilities and the phase behaviors of solid ammonia phases I and IV more accurately, we calculate their Gibbs free energies at CCSD(T)/CBS levels. Figure 6 shows the Gibbs free energy surfaces of solid ammonia phase I and phase IV from 0.5–6.0 GPa, based on the CCSD(T)/CBS theory. From Fig. 6, the free energies of phase I are lower than phase IV when the pressure <1.17 GPa and the temperature <210 K. With the pressure increasing, the two surfaces generate a crossing line and phase IV became a more stable phase after that. Therefore, the crossing line is the phase transition line of solid phases I-IV.

### Gibbs free energy difference

**Figure 7:**
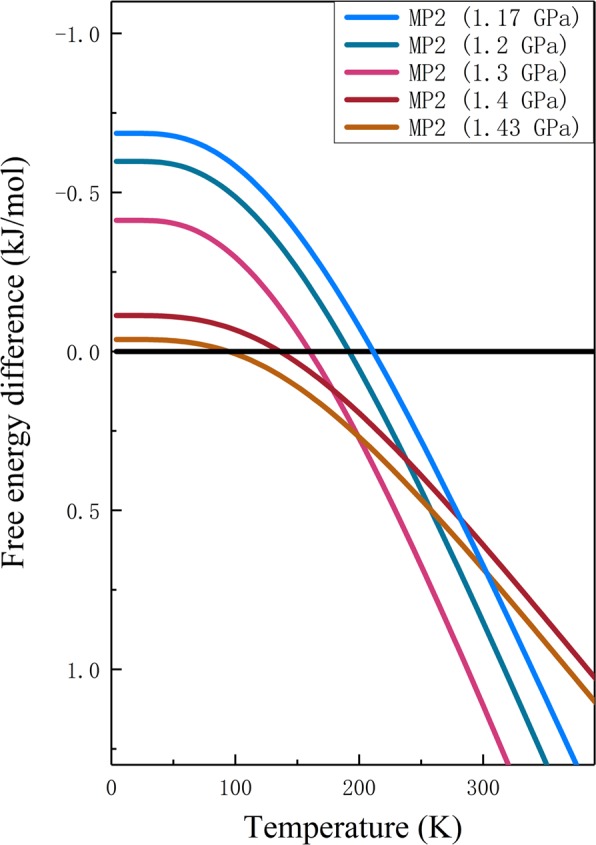
Gibbs free energy difference between NH_3_ phases IV and I (free energy of the phase IV minus that of phase I). The positive value means that phase I is more stable. The calculations are performed at the CCSD(T) level with CBS extrapolations.

Figure 7 shows the Gibbs free energy differences between solid ammonia phases I and IV at different pressures computed at the CCSD(T) level with CBS extrapolations. As shown in Fig. 7, the positive value denotes that the Gibbs free energy of phase I is lower and the structure is more stable than phase IV. Specifically, the Gibbs free energy difference equals 0 at 1.17 GPa when the temperature gets to 210 K. When the pressure increases to 1.2 GPa, the transition temperature decreases to 190 K accordingly. Further increasing the pressure to 1.3, 1.4, and 1.43 GPa, the phase transitions between NH_3_ phases I and IV take place at 162, 134, and 94 K, respectively.

### Phase transitions

**Figure 8:**
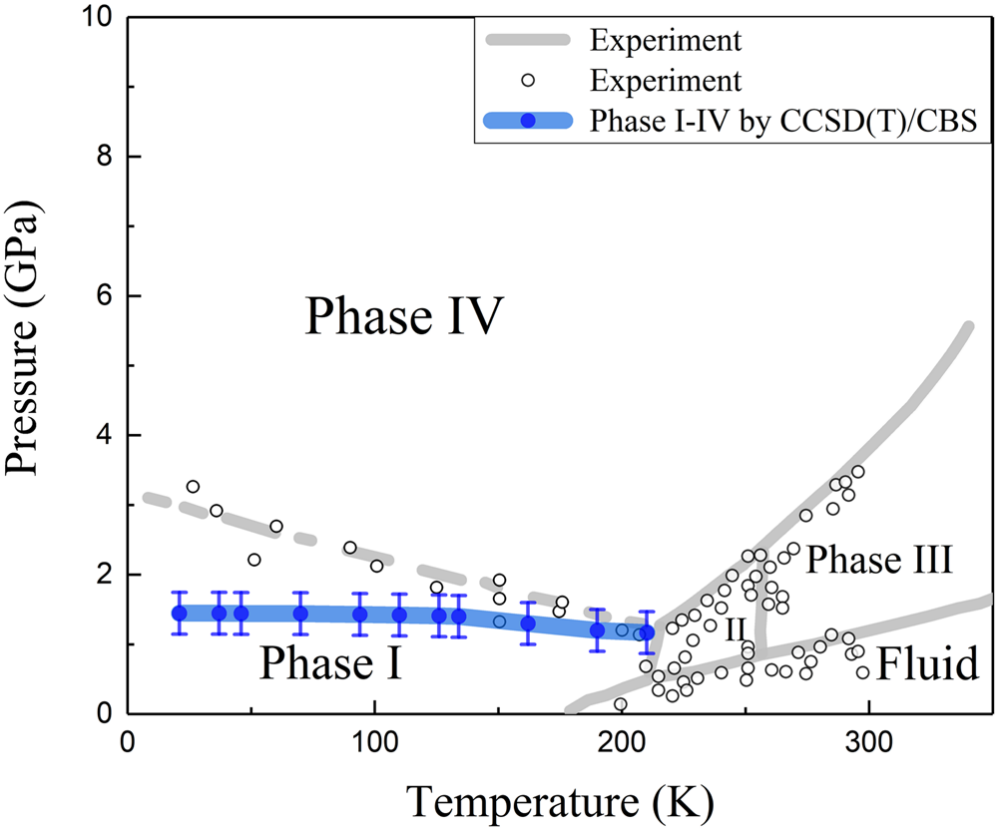
The phase diagram of solid ammonia, where the solid grey lines and dots are the confirmed phase boundaries in laboratory^6,10–12,59^, the dashed line is the speculated phase boundary between phases I and IV^10^, while the blue line and dots are the calculated phase boundary between solid ammonia phases I and IV using CCSD(T)/CBS, the error bar is set to ±0.3 GPa.

Figure 8 shows the phase diagram of solid ammonia within the pressure range of 0–10 GPa, where the solid grey lines are the observed phase boundaries between different phases of solid ammonia^6,10–12,59^, the dashed grey line is the speculated phase boundary between phases I and IV in laboratory^10^, and the blue line and dots denote the predicted transition boundary of NH_3_ phases I and IV using CCSD(T)/CBS. As shown in Fig. 8, the phase transition boundary of phases I and IV was measured by Gauthier *et al*.^10^. However, they did not observe the boundary clearly due to the limited experimental conditions and just marked it out in dash line. Such speculation is still in use today, but needs to be verified. The present calculations confirm the work by pioneers that the solid ammonia phase IV has a space group of *P2*_1_2_1_2_1_ and predict the similar phase transition boundary between phases I and IV with Gauthier *et al*.’ s speculation^10^ within small pressure variations. As one can see from Fig. 8, the predicted phase transition pressure between phases I and IV is 1.17 GPa at 210 K, with a small transition pressure change as temperature decreases.

## Conclusions

Given the needs to discover high-density solid energy materials and the high cost of performing experimental solid phase screening, many studies have focused on the computational structure predictions. The recent blind crystal structure tests have been made in predicting possible crystal structures and properties from first principles, but the truth is that the small changes in the free energies can completely alter the structural alternation of polymorphs, and therefore predicting the thermodynamic conditions under which the polymorphs can be produced remains a challenge. In this study, we calculate the structures, lattice constants, Raman spectra, Gibbs free energies of solid ammonia phases I and IV at high pressures, and thus predict their phase transitions from first principles based on the MP2/aug-cc-pVDZ and CCSD(T) theories with CBS extrapolations. We reproduce quantitatively the lattice constants, equation of state, and the Raman bands of ammonia phases I and IV, which confirm the experimental data. The predicted results also support Gauthier *et al*.’ s speculation that the phase transition of NH_3_ phases I and IV occurs between 1 to 2 GPa and below 210 K, with a small temperature variation. The limitation of the proposed calculation is that it only considers the harmonic approximation of phonon contribution when calculating the Gibbs free energies, and the present work only applies to molecular crystals and small molecule systems. Future work includes improving the accuracy of free energy calculations by considering the anharmonic approximation and further refining the method’s applicability to ionic crystals and macromolecules.
